# The evolution of the liver transplant candidate

**DOI:** 10.3389/frtra.2023.1178452

**Published:** 2023-07-03

**Authors:** Angus Hann, James Neuberger

**Affiliations:** ^1^ The Liver Unit, Queen Elizabeth Hospital Birmingham, Birmingham, United Kingdom; ^2^ Centre for Liver and Gastrointestinal Research, Institute of Immunology and Immunotherapy, University of Birmingham, Birmingham, United Kingdom

**Keywords:** liver, transplant, cirrhosis, acute liver failure, organ allocation

## Abstract

The first successful human liver transplant (LT) was done over 60 years ago; since the early pioneering days, this procedure has become a routine treatment with excellent outcomes for the great majority of recipients. Over the last six decades, indications have evolved. Use of LT for hepatic malignancy is becoming less common as factors that define a successful outcome are being increasingly defined, and alternative therapeutic options become available. Both Hepatitis B and C virus associated liver disease are becoming less common indications as medical treatments become more effective in preventing end-stage disease. Currently, the most common indications are alcohol-related liver disease and metabolic associated liver disease. The developing (and controversial) indications include acute on chronic liver failure, alcoholic hepatitis and some rarer malignancies such as non-resectable colorectal cancer liver metastases, neuroendocrine tumours and cholangiocarcinoma. Candidates are becoming older and with greater comorbidities, A relative shortage of donor organs remains the greatest cause for reducing access to LT; therefore, various countries have developed transparent approaches to allocation of this life saving and life enhancing resource. Reliance on prognostic models has gone some way to improve transparency and increase equity of access but these approaches have their limitations.

## 1. Introduction

**Figure 1:**
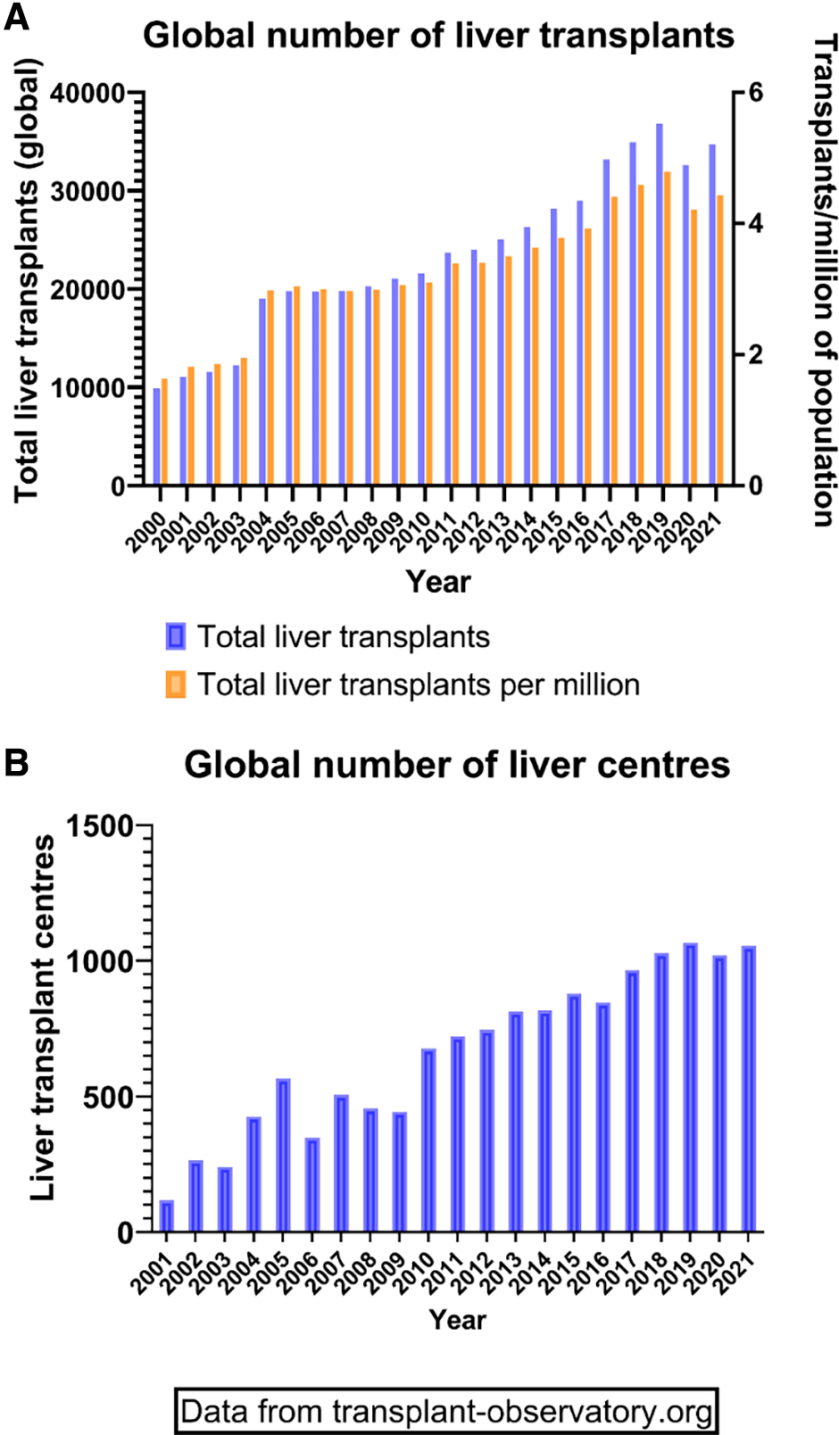
Global transplant activity. (**A**) Demonstration of total liver transplants (left *Y* axis) and transplants in relation to the population size (right *Y* axis) since the year 2000. (**B**) Total number of active liver transplant centres around the world since 2000.

The founding father of liver transplantation, Thomas Starzl, identified 5 interlocking themes that led to success in human liver transplantation, and its transition from an experimental procedure with a high perioperative mortality and poor long-term survival to the routine procedure of today (1) (Table 1). These themes continue to be developed and contribute to the ongoing improvements and continued success of liver transplantation today (Table 1). It has now been 60 years since the first liver transplant was attempted in a human, and this journey has included both successes and failures. However, both have led to accrued knowledge, understanding and subsequent refinements in practice (Table 2) Liver transplantation is now available in approximately 1,200 centres around the globe (Figure 1A), with over 40,000 procedures being undertaken annually (Figure 1B).

**Table 1:** Five themes leading to the development of successful human liver transplants [from Starzl and Fung (1)].

Theme	Description
I	Began 1n 1958–59 with canine studies of then theoretical hepatotrophic molecules 1n portal 5enous blood. These studies 1dentified that 1nsulin was the main hepatotophic factor and 1dentified the 1mportance of the portal 5ein.
II	The development of liver and multivisceral transplant models which developed 1n parallel with Theme 1.
III	Is the development of successful immunosuppression which included azathioprine, prednisone, and ALG.
IV	Is the development of newer immunosuppressive agents and better understanding of the mechanisms of rejection and tolerance which allowed for which include cylosporine (1979), tacrolimus (1989) and more recently, mycophenolate and sirolimus/everolimus (1999).
V	Represents the human aspects, including the ethical and legal issues surrounding both organ donors as well as recipients.

**Table 2:** Some key dates in the field of liver transplantation.

Date	Event	Pioneers
1955	First article 1n literature about auxiliary liver transplant	Welch
1959	Models of canine total hepatectomy and replacement	Staudacher, Welch, Starzl, Cannon
1963	Introduction of corticosteroids/azathioprine	Starzl
1963	First attempt of human liver transplant (survival up to 3 weeks)	Starzl
1967	1 year survival of human liver transplant recipients	Starzl
1979	Introduction of cyclosporine	Calne, White
1981	Reduced size liver graft	Bismuth, Houssin
1983	Combined liver kidney transplant	Margreiter
1984	Combined heart and liver transplant	Starzl
1987	Combined heart-lung liver transplant	Calne, Wallwork
1989	Combined liver intestine transplant	Starzl
1989	Introduction of tacrolimus	Starzl
1989	First split liver transplant	Pichlmayr
1990	Living donor transplant	Raia, Strong
1993	Baboon to human liver transplant	Starzl
1995	Domino liver transplant	Furtado

In order to do the greatest justice to all those in need, both recipients and donors need to be carefully selected. The aim of a liver transplant is to increase survival and/or improve the quality of life. Identifying those conditions which give a survival benefit with transplant as the treatment is straightforward in the majority of instances and can be determined by comparing post-transplant survival with transplant free survival within a cohort. However, selecting recipients at the individual level is far more challenging as it requires consideration of the operative risks and the optimal timing of transplant. If left until too late a stage in the disease, they could be too sick to survive the procedure or the tumour may become too advanced to have an acceptable outcome; conversely, if transplanted too early, survival may be reduced and the recipient may experience complications that would not have occurred if they had persisted with their native liver. Widespread acceptance of emerging transplant indications is highly reliant on the appropriate definition of the recipient population with that benefit being accurately defined, reviewed and, where appropriate, revised. Furthermore, access to grafts varies between programs and between jurisdictions and this will undoubtedly influence which individuals are transplanted and when. This review will further describe some of these concepts, with a particular focus on how transplant indications, recipient selection and allocation have changed over time.

## 2. Liver transplant: from experimental procedure to accepted service

The initial attempts at human liver transplantation were characterised by very poor outcomes: out of the transplant recipients, grafted between 1963 and 1964, none survived more than 23 days (2). The youngest of this small group was a 3-year-old with biliary atresia, whom died on the day of surgery from uncontrollable bleeding (3). The remaining seven, grafted in Denver, Boston and Paris, were transplanted for cancer (including two with colonic metastases) and the main causes of death were sepsis, bleeding and pulmonary emboli. These outcomes led to a 3-year moratorium in clinical practice; liver transplantation was re-started in 1967 by Starzl in Denver, and the following year a programme was started in Cambridge, UK, led by Sir Roy Calne.

The outcomes in the first years after the inception of this procedure, were very disappointing by today's standards. Thus, of the first 25 recipients in the Cambridge series, transplanted between 1968 and 1971, only 1 survived more than 1 year. This female patient was transplanted for primary liver cell cancer, and ironically would not meet current transplant criteria. Only 6 of these 25 recipients lived for more than 3 months. It is a great tribute to the courage, perspicacity, tenacity and dedication of these visionary transplant teams that the clinical and scientific teams continued to persevere. Furthermore, these pioneers managed to improve all aspects of the procedure, from selection of recipients and donors, to better retrieval, preservation, anaesthetic and surgical techniques. The understanding of the immunological, microbiological, haemostatic and physiological processes these individuals were able to achieve without the advanced technologies of modern day is remarkable. Since those early days, transplantation has expanded dramatically, as shown by the increase in both the number of patients transplanted and the number of transplant centres (Figure 1).

Based on a small number of pioneering transplant centres (and the relevant clinical and scientific personnel), the first National Institutes of Health (NIH) Consensus Conference was held in 1983 (4). In this forum, Starzl and colleagues argued that liver transplantation had met the criteria necessary for it to be an accepted intervention. The rationale for this statement was based on (1) The procedure was within the capability of more than the “ocasional surgeon” (2) The indication for the procedure were clear, and (3) The results were good enough to justify the effort and expense (4). The outcomes of 296 patients grafted between March 1963 and April 1983 were reviewed. Up to the end of 1979, 170 had been grafted; 56 (33%) of the recipients lived for at least 1 year; 26 survived 5 years and 6 survived 10 years. Death occurred mainly in the first 3 months and was attributed to the advanced stage of disease of recipients, technical surgical issues, the use of damaged liver grafts, the inability to control rejection, and a variety of infections. Most of the deaths in the first half of the second year (Table 1) were due to chronic rejection. During this period, immunosuppression was with azathioprine and prednisone, and induction with anti-lymphocyte globulin in most cases. In the subsequent period, 1980–1982, of the 40 recipients with a follow-up extending to 38 months at the time of publication, the 1-year survival was 70%. Causes of death after 1 year included recurrent cholangiocarcinoma, recurrent Budd-Chiari syndrome, and chronic rejection (now classified as ductopenic rejection). Improved outcomes were attributed to the use of cyclosporin and corticosteroids.

During these early years, it had also become clear that the clinical state of the recipient had a major association with outcome. The 6-week survival rate was 42% for the 26 patients taken to transplant from the intensive therapy unit (ITU) in comparison with 68% for the 63 patients who were managed as outpatients. On the basis of this limited data, the Conference concluded that liver transplantation could be considered for most patients who are near death with liver disease and should be considered as a service provision rather than an experimental procedure. The panel did not address the issues of who should pay for liver transplants or of how many donor livers would be needed if more transplantation centres were established (5).

Advances in adult liver transplantation have been mirrored in paediatric transplants. The lower age of recipients has been falling so now babies only a few months old are being successfully grafted (6). Further discussion of liver transplantation in the paediatric patient population is beyond the scope of this review.

## 3. Evolution of indications for liver transplant

**Figure 2:**
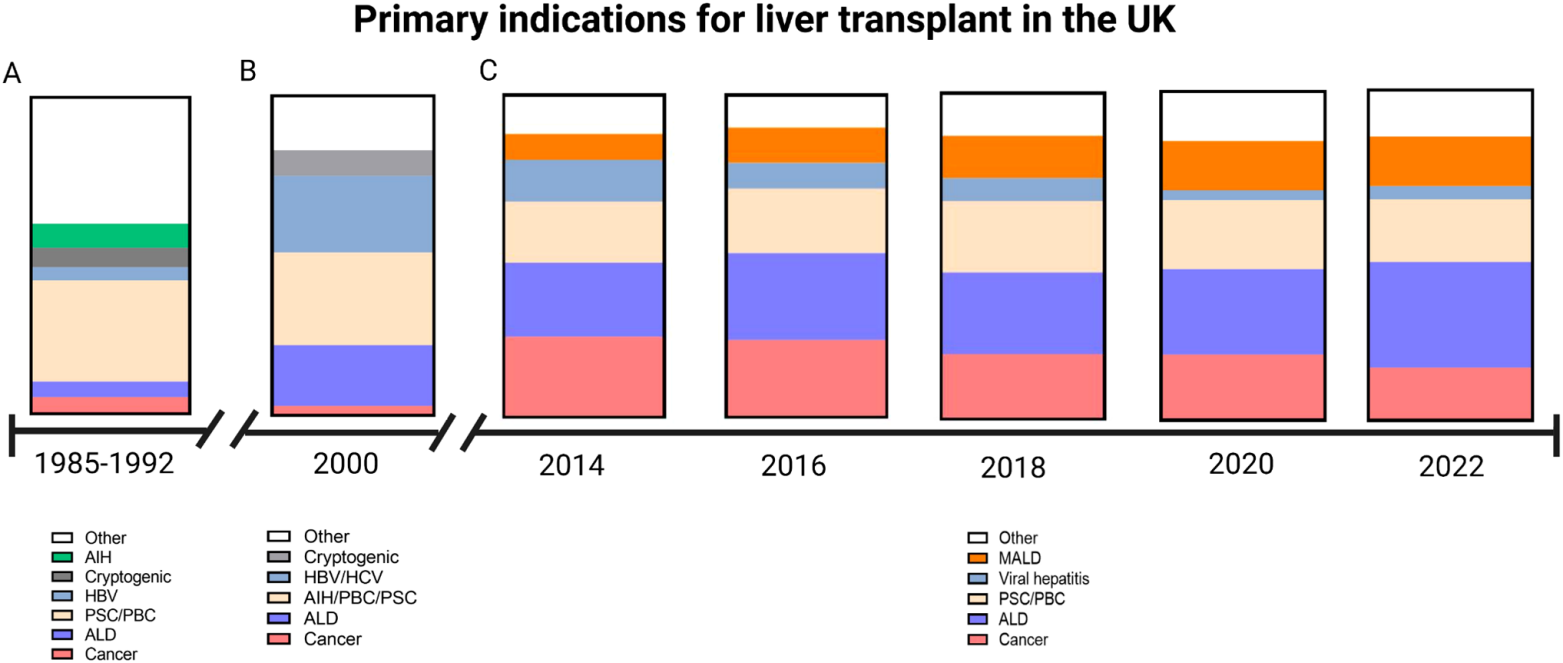
Graphical demonstration of primary indications for liver transplant within the UK. (**A**) Indications between 1985 and 1992 as reported by Devlin et al. During this period there was a low number of patients transplanted for alcoholic liver disease (ALD) as the primary indication (Source Devlin et al. Gut 1999 45:VI1–VI22). (**B**) In the year 2000, a greater proportion of patients were transplanted for ALD and viral hepatitis (Source: Prince et al. 2002. Postgraduate medical journal 78:135–141). (**C**) Data obtained from the annual National Health Service annual reports on liver transplantation show a decline of cancer as the primary indication and an increase in metabolic associated liver disease (MALD) (Source: https://www.odt.nhs.UK/statistics-and-reports/organ-specific-reports/).

In the early days of liver transplantation, indications included cancer, cirrhosis and acute liver failure. Thus, of the 227 liver transplants carried out by the Cambridge/Kings College Hospital team between 1968 and 1985, around one third were for malignancy (primarily hepatocellular carcinoma but cholangiocarcinoma, carcinoma of the hepatic ducts and hepatic metastases were also included); of those with non-malignant liver disease the majority were for end-stage cirrhosis (primarily primary biliary cholangitis, autoimmune hepatitis, cryptogenic cirrhosis and alcoholic liver disease). There were 13 cases of Budd-Chiari syndrome, 6 of acute liver failure and 16 biliary atresia and neonatal hepatitis. Over the next four decades, the indications have changed (see Figure 2). The advent of better treatment options for liver cell cancer, the effective treatments for control of Hepatitis B virus and for cure of Hepatitis C virus, means that these indications are becoming less common and being replaced by metabolic associated fatty liver disease (also termed non-alcoholic fatty liver disease) and alcohol-related liver disease.

As many of the technical issues of transplantation have been resolved, clinicians are looking at new indications. One of the greatest challenges in solid organ transplantation is the discrepancy between the number of patients who would benefit from transplantation (in terms of length of life and/or quality of life) and the number of organs suitable for transplantation. This means there has to be rationing, which has resulted in jurisdictions developing rules that are not only transparent but based on a combination of the sometimes competing requirements of utility, justice, equity, benefit and fairness (7). Thus, some people who would benefit from transplantation are denied access because they would not benefit *enough*.

There also needs to be a clear process of defining futility when the benefits of transplantation become insufficient to justify access to a rationed resource. In 1999, a small group defined that LT should be considered only when there was a greater than 50% probability that the patient would be alive 5 years after transplant with a quality of life acceptable to the patient (8). These arbitrary guidelines have remained as a useful guide to avoiding futility. It is of interest that most jurisdictions require the same criteria for recipients of living donor organs as for deceased donor organs, even though the impact of the organ shortage does not directly affect the living donor organ recipient. It is beyond the remit of this article to argue whether this restriction is appropriate.

While there is no question that there need to be transparent selection and allocation policies, underpinned by a legal framework, too rigid selection and allocation policies will inhibit development of new indications. The development of new indications and removing some contra-indications is challenging since innovation is required to advance and also allow equity of access across a broader group of patients who night benefit from LT (9). Thus broadening indications must be paralleled by either increasing the availability of organs or removing some current indications, otherwise the waiting list mortality will rise. Thus, different countries have different need and availability of organs and so indications will vary.

It is against this background of limited life-saving resources that evolving indications must be considered.

Below we outline some of the changing indications.

### 3.1. Evolving indications

#### 3.1.1. Alcohol-related liver disease and alcoholic hepatitis

Alcohol related liver disease (ARLD) has become one of the commonest indications for LT. However, in the early years of transplantation, ARLD was considered a poor indication for liver transplantation because of the high risk of infection and because of uncertainty about the likelihood of abstinence (10). However, just over a decade later, the same authors were arguing in favour of LT for those with ARLD, stating outcomes were good and recidivism rates low (11). During the ensuing decade, there was considerable controversy in the professional and lay press about the ethical and clinical issues surrounding the use of scarce resources for those with ARLD. Initially, the public were against the use of organs for those with ARLD (12) but, over time, opinion is changing and is becoming more supportive (13). Concerns that those transplanted for ARLD would return to a damaging pattern of alcohol use or become non-compliant have been only partly resolved as data shows graft loss from recurrent alcohol use is much less than feared and less than graft loss from recurrent disease for other indications. Public attitudes to the use of donated organs is important as transplantation is uniquely dependent on public support. When this is lost (as seen in Germany recently when there was evidence of clinicians falsifying patient data thus giving selected patients an unfair advantage), donation rates fall dramatically. However, adverse publicity surrounding alcohol misuse by transplant candidates has no demonstrable impact on organ donation or consent rates (14). Criteria have now been developed and agreed for assessing, supporting and monitoring those with ARLD; the 6-months' requirement for abstinence, still required by some units and jurisdictions has little evidence base or predictive value for future abstinence.

The role of transplantation for those with alcoholic hepatitis has evolved in recent years. Initially considered an absolute contraindication for transplantation, the pivotal study by Mathurin showed that good outcomes could be obtained in highly selected individuals (15). However, outcomes are not as high as in other indications and a recent prospective study did demonstrate that early transplant is associated with a greater risk of alcohol use (16). Indications and timing are becoming clearer; however, concerns about the selection of patients remain: outcomes of those who were listed for transplantation showed a significant but small proportion will recover without transplant. Current medical treatments for alcoholic hepatitis are improving. Since many patients are sick and often encephalopathic, there is little time and opportunity to identify those who are likely to return to a damaging pattern of alcohol use or put in place supportive measures to ensure abstinence. In our view, there is certainly a place for LT in some of those with alcoholic hepatitis but criteria for the selection of appropriate candidates is still evolving.

#### 3.1.2. Cholangiocarcinoma

Cholangiocarcinoma (CCA) was not an uncommon indication for LT in the early years but in the 1990s CCA became a contra-indication as outcomes were generally very poor, with 5-year survival rates around 30% with death usually related to recurrent (or more accurately persisting) disease. Developments in understanding the natural history and classification of cholangiocarcinoma and the introduction of the “Mayo protocol” have resulted in some jurisdictions allowing the use of LT for this indication. The Mayo protocol, first introduced in 1993, is aggressive, consisting of a 3-week course of external beam radiotherapy and continuous infusion of 5-Fluorouracil, followed by a 2-week course of brachytherapy, capecitabine followed by an abdominal laparotomy and staging. If there is no evidence of spread, these patients may be listed for transplantation.

CCAs may be classified into intrahepatic CCA (iCCA), perihilar CCA (pCCA), and distal CCA. As with HCC, surgical resection is the primary treatment where possible and there is no advanced parenchymal liver disease but, even with greater awareness and possibly better and earlier diagnosis, up to 70% of CCAs are inoperable at the time of diagnosis. Improvement in patient selection criteria and neoadjuvant treatment protocols have improved outcomes for otherwise inoperable pCCA patients. Patients with iCCA less than 2 cm on cross sectional imaging seem to fare best. Treatments ad protocols are evolving (17).

#### 3.1.3. Colorectal metastases (CRM)

Colorectal metastatic disease was, in the early era of LT, an occasional indication for liver transplantation but soon became a contra-indication. However, with greater understanding of the natural history of CRM and the ability to use molecular profiling, it is becoming less complex to define a small cohort of those with CRM who may benefit from liver transplant (18). However, it should be stressed that the numbers of patients transplanted for CRM remains relatively small and a recent meta-analysis identified only 48 patients transplanted for CRM and did report a slight survival benefit for LT compared with palliative therapy, with almost all of the deaths related to the cancer (19).

#### 3.1.4. Neuroendocrine tumours

The role of LT in those with unresectable neuroendocrine liver metastases remains uncertain. A recent meta-analysis concluded that surgical resection is the best treatment when metastases are resectable, although liver transplantation shows good results for patients not eligible for surgery (20). However, recurrence rates are relatively high (around 35%).

### 3.2. Other changing indications

*Budd-Chiari syndrome* was initially considered a very good indication for LT (21). However, with greater understanding of the natural history and pathophysiology and the advent of improved radiological interventions such as thrombolysis and use of transjugular intra-hepatic portal systemic stents (TIPS), LT is being used much less commonly (22).

#### 3.2.1. HIV infections

The advent of highly effective anti-retroviral infection treatment has allowed those living with HIV and with ends-stage disease from conditions such as HCV infection can now be offered LT. Indications have been agreed and outcomes are improving (23). This is of particular relevance in countries such as South Africa, where the prevalence of HIV infection in the 15–49 year old group is reported to be close to 18% (24).

#### 3.2.2. Hepatocellular carcinoma

As previously described, liver transplantation has been used as treatment for hepatocellular carcinoma since the inception of the procedure. However, the criteria for transplant eligibility have evolved significantly over time. This has been in an effort to minimise early death from cancer recurrence and optimise graft utility. A seminal publication in 1996 by Mazzafero et al. reported a number of HCC tumour characteristics (known as the Milan Criteria) that were associated with a favourable outcome (25), as prior to this the survival outcomes of less selected recipients with more advanced disease was poor (26). In many countries, including the UK, the Milan Criteria remain in place today for selecting appropriate patients for the waitlist. However, a criticism of the Milan criteria is that it may be too conservative and additional criteria have been reported (27). In 2001, Yao et al. reported a 75% post-transplant 5 year survival in a cohort of patients with a larger maximal tumour size and incorporated the idea of a total tumour diameter for those with multiple lesions (28). Furthermore, these authors suggested biological markers such as alpha feto-protein (AFP) may be of benefit. These findings are referred to as the University of California San Francisco (UCSF) criteria. In an effort to refine the prediction of outcome further, the Metroticket 2.0 model was published in 2018 (29). In contrast to the previous models, this system considered deaths as non-cancer related or HCC related and therefore analysed the data in an effort to specifically predict the latter. The Metroticket 2.0 system has failed to replace the more widely adopted Milan and CSF criteria. In recent years, exciting advances have been made in the area of downstaging patients with tumours exceeding the accepted criteria to within the limits accepted for transplantation. Combinations of locoregional, systemic and immunotherapy have been applied in this scenario and have shown promising results (30–32).

#### 3.2.3. Simultaneous organ transplantation

Over time, multi-organ transplants are becoming more common. Liver combined with kidney remains the most common multi-organ transplant. In the US, simultaneous liver kidney transplants increased from 1.7% of all liver transplants to 9.9% by 2016 (33). This increase is likely to be driven in greater part by the MELD-based allocation system which prioritises those with renal impairment and hepato-renal syndrome. Other indications for combined liver-kidney transplants include dual pathology such as polycystic disease. Other combinations of organs include liver-heart, liver-lung and multivisceral abdominal organ transplantation The first successful liver-heart transplant was performed in 1984 in Pittsburgh (34). Between 2000 and 2015, the number of these procedure performed each year in the U.S. has increased but overall remains low (approx. 30 in 2015). The most common indication for the liver transplant in this procedure is familial amyloid polyneuropathy, followed by cardiac cirrhosis (35). These recipients obviously represent a highly selected group, but acceptable survival is achievable (35). The majority of these procedures have been performed with the two organs separated, however enbloc heart-liver transplant maintaining the suprahepatic inferior vena cava was described by Hill et al. in 2012 (36). This procedure is increasingly utilised in the situation of liver cirrhosis and failing fontan physiology (37). Lung-liver transplants are performed even less frequently than heart-liver transplants, with less than 12 being performed in the U.S in 2018 (38). The main indication for this procedure is cystic fibrosis (39). In both these scenario (heart liver or lung liver), the rationale of transplanting both organs at the same time rather than sequentially is that the recipient would survive each in isolation. Liver-intestine or multivisceral (Liver-intestine-stomach) abdominal organ transplantation is performed for intestinal failure and its associated complications. The complication that most frequently necessitates intestinal transplantation with the liver is intestinal failure associated liver disease (IFALD) (40). An additional indication for this combined procedure is diffuse splanchnic venous or arterial thrombosis (41). The intestine containing graft is the most immunogenic of all organs that are currently being transplanted and severe rejection, infection and post-transplant lymphoproliferative disease are frequent. European outcome data suggests that in adults undergoing liver-intestine transplantation, graft survival is approximately 50% at 2 years follow up (41). In contrast to other areas of intestinal failure management, transplanting the small bowel remains a challenge and perfecting this will undoubtedly provide a great deal of benefit to numerous patients.

## 4. Evolution of the transplant candidate risk profile

As liver transplantation has evolved, the proportion of candidates with extra-hepatic risks have increased (42). For example, in the US the proportion of patients aged 65 years or over rose from 13.5% in 2009 to 28.9% whereas the proportion of listed candidates aged between 50 and 64 years fell from 64.3% to 51% (43). As transplants candidates are becoming older and, as a consequence, are more likely to have comorbidities such as cardiovascular, pulmonary and cerebrovascular disease as well as malignancy. Furthermore, as the indications evolve, more candidates with metabolic associated liver disease and alcohol related liver disease are referred and both indications are associated with risk factors. There are well developed algorithms for assessing the cardiovascular status of liver transplant candidates (44) which set out guidance for evaluation but provide little information as to what constitutes contra-indication.

As experience has accrued, it became evident that it was not just recipient factors in isolation that related to post-transplant outcome, but rather the donor/recipient match (45). This has been further investigated by several groups and scoring systems such as the survival outcomes following liver transplantation (SOFT) and Balance of Risk (BAR) (46, 47). An issue with both these scores are that they are generated from the United Network of Organ Sharing (UNOS) database of patients transplanted, therefore provide little insight into the dilemma whether someone should be listed in the first place. Further concerns relate to these large national data bases with variation in definitions between centres, variation in laboratory values and incompleteness of data; these analyses can be based only on those data collected and some essential data, which may be subjective, are not collected. Models are necessarily based on historical data and may not reflect time-dependent variables. Models developed in data in one jurisdiction may not be applicable to other countries (as we have seen with MELD and UKELD), Furthermore, many models do not give confidence intervals and not all are externally validated, On the other hand, the registry data give the benefit of large numbers. Many of these concerns are inherent in the use of registry data and do not invalidate the reports or detract from their great value, but should be born in mind when applying them prospectively to an individual.

The SOFT score, published in 2009, used univariate and subsequently multivariate modelling to identify donor, recipient and operative factors that were associated with 3-month mortality risk (47). On univariate analysis of recipient factors, the highest odds ratio for mortality was the recipient being on life support pre-transplant or undergoing a retransplant. Two scores were reported, the SOFT and Preallocation-SOFT (P-SOFT), with the latter not including donor factors (47). The final score incorporated 18 factors, 6 of which related to the donor, and another was graft cold ischaemic time (CIT). The BAR score was published in 2011, and aimed to provide a more simplified and practical score for donor/recipient matching (46). This score was limited to six factors, two of which were the donor variables of age and CIT. In the cohort used to calculate the score, split grafts and grafts from circulatory death donors (DCD) were excluded which limits its applicability. Although designed with good statistical methods, these scores have failed to make it into mainstream allocation systems.

## 5. Marginal grafts and machine perfusion technology

The constant shortfall of liver grafts, in relation to the list of recipients in need, has made clinicians utilise donors and grafts with features that had been considered suboptimal by the “traditional” metrics. The use of non-heart beating donors (later referred to as DCD), elderly donors and steatotic livers are just a few examples of areas were boundaries have been progressively pushed. The concept of brain death was introduced in 1968 and more universally accepted in the years following (39, 48). Therefore, all human liver grafts used during the introductory phase of this procedure by Starzl and Calne were not only DCD grafts (49), but in many cases uncontrolled DCD (Maastricht IV) by todays standards (50). In general, a marginal organ is one that has factors associated with an increased risk of both early and late graft loss (51). The term marginal graft is becoming replaced by the term “extended criteria donor” (ECD) graft. Both terms have limitations in that, while the term implies increased risk to the recipient, the risks will vary from increased risks of primary non-function, delayed function or other graft pathologies, or carry increased risk because of the risk of donor transmitted diseases,.

The reintroduction of DCD liver transplants occurred in the 1990s due to the excessive demand for organs (51). The early results from Pittsburgh, in a patient series between 1989 and 1993, showed unacceptably high rates of graft loss within 3 months for both controlled (50%) and uncontrolled (83%) DCD. Despite these results, the practice continued and results from D'Allesandro et demonstrated a PNF rate of 10.3% in controlled DCD transplants as opposed to 1.3% for DBD (52). Although these authors demonstrated a greater risk of PNF and biliary strictures in DCD transplants in comparison to DBDs, it was not prohibitively high and DCD transplants continued. As more experience with DCD liver transplants was gained, it became apparent that the duration of time from withdrawal of life sustaining treatment to cold preservation had a large impact on outcomes (53), and livers with a prolonged functional warm ischaemia (>30 min) should be transplanted with caution (54). Thrombosis of the biliary vascular plexus during this period has been considered as the cause of biliary injury, and attempts to reverse this with thrombolysis at different points of retrieval and implantation have been trialled (55, 56). In the recent decade, DCD grafts have achieved similar outcomes to DBD grafts and this may be due to more precise selection of the appropriate recipients. Patients with less severe liver failure, particularly those listed for HCC rather than MELD/UKELD, have comprised a larger portion of DCD recipients in the studies that have reported improving DCD outcomes (55, 57). This is reflected in current practice recommendations whereby recipients of DCD grafts are generally less surgically complex and have lower MELD scores, making them more physiologically tolerant of an early period delayed graft function. Overall, an individuals survival has been shown to be greater with the acceptance of a DCD graft in comparison to only consideration of a DBD (58). The further influence normothermic regional perfusion (NRP) is having in DCD liver transplantation is described later. Furthermore, any increased morbidity or mortality associated with marginal grafts must be balanced against the reduction in risk of death awaiting transplant.

Graft steatosis is a major contributor to non-utilisation of donor livers. Large droplet fat vacuoles within the hepatocyte are well known to make the donor liver less tolerant of the ischaemic periods and causes a greater ischaemia reperfusion injury. This manifests clinically as severe early allograft dysfunction or primary non function (59). Graft steatosis has long been recognised not only as a risk for poor outcome, but as a factor that interacts with other donor and preservation characteristics (such as cold ischaemic time) to magnify the risk (60). Over the course of time, it has been demonstrated that our actual assessment of graft steatosis is imperfect (61, 62). In an approach similar to DCD grafts, the conventional approach is to select recipients for steatotic grafts to be more robust so they can withstand an often severe post reperfusion syndrome and stormy early post-operative period. In a recent US registry publication by Jackson et al, the overall survival benefit with receiving a steatotic graft was actually greater in those with more severe liver disease (MELD 35–40) (63). The most likely explanation of this finding is that mortality without transplant is exceptionally high in this group, and this exceeds the early perioperative risks.

Machine perfusion technology has been having an increasing role in liver transplantation over the last decade. These comprise both in-situ (NRP) and ex situ [Normothermic machine perfusion (NMP) and hypothermic machine perfusion (HMP)] techniques. NRP is used in the setting of DCD donation and involves a period of in-situ resuscitation via an extracorporeal membrane oxygenation circuit after the donor warm ischaemic period. This method allows graft viability to be assessed whilst the liver remains in the donor, and replenishes the energy stores before the second insult of cold ischaemia. Although the existing evidence is only from large cohort studies rather than randomised controlled trials, this technique undoubtedly reduces the incidence of non-anastomotic strictures after DCD transplantation (64–66). Furthermore, the improved function of NRP preservation has made DCD transplant a for even high risk retransplant candidates (65). Both NMP and HOPE provide oxygenated perfusate at either 37°C or 4°C whilst the liver is outside the body. NMP allows viability assessment of grafts with marginal features, can safely prolong the preservation period and has been demonstrated to safely facilitate transplant into high risk recipients (67, 68). HMP has demonstrated the greatest benefit for DCD grafts, as a randomised controlled trial found a reduction in non-anastomotic strictures with DCD preservation (69). Machine preservation strategies have changed the conventional paradigm of graft-recipient matching as they mitigate some of the risk factors associated with poor outcomes. This has allowed the suitable donor pool to be expanded for a number of transplant recipients that had narrowed opportunities.

## 6. Evolution of allocation policies

Organs from deceased donors are considered a national resource. As liver transplantation has become a routine procedure available widely, the gap between the number of patients who need a liver transplant and the number of suitable organs available for transplant has increased, and become more transparent. Despite a number of initiatives to increase organ donor availability, this gap remains a major problem, meaning that a resource that is life saving and life enhancing must be rationed and therefore jurisdictions have developed methodologies to provide an objective and transparent approach to organ allocation.

The evolution of organ allocation in the United States has evolved over four decades (70). 1984 saw the establishment of UNOS, the National Organ Transplant Act (NOTA). UNOS manages OPTN (The Organ Procurement and Transplantation Network) and is responsible for maintaining the national database for organ transplants and for allocating donated human organs. In 2000, the “Final Rule” was published by the United States Department of Health and Human Services establishing federal regulations on OPTN policies including listing requirements, organ procurement, identification of an organ recipient, allocation of donated organs, designated transplant program requirements, reviews, evaluation and enforcement of transplant programmes. Amongst the roles set for UNOS are developing and implementing rules for the equitable distribution of donated organs. NOTA requires UNOS to establish medical criteria for the allocation of organs which should include measures to ensure equity and justice, this included the need to agree medical criteria for determining suitable transplant candidates' access to the waiting list and set priorities based on objective and measurable criteria, so that the most urgent are offered organs first.

In the US, priority is given to those who have acute onset liver failure and are deemed not likely to survive more than a few days without an OLT (status 1A) and those who are very sick, chronically ill pediatric patients with cirrhosis who are younger than 18 years of age-pediatric population (status 1B).

Assessment of severity: prior to 2002, the Child-Turcotte-Pugh model was used to assess the severity of disease. This model was largely empirical and designed to assess prognosis after surgery in those with advanced cirrhosis. Because this model was developed by clinicians, it became widely used, despite its lack of rigorous development and validation. However, criticism arose not only on the concerns about the statistical validity, but the use of subjective components (such as ascites and encephalopathy).

In 2002, the MELD model was introduced (Model for endstage liver disease) (71). This was developed by Kamath and colleagues as a tool to assess prognosis of those with advanced liver disease undergoing variceal surgery. The model was based on objective laboratory criteria only and was validated in many other situations and showed a reasonably good predictive ability. There have been a number of concerns and suggested modifications such as inclusion of sodium levels in the model and the MELD-Na score was agreed in 2014 and implemented 2 years later, concerns that the use of serum urea will disadvantage females and that non-hepatic factors (such as haemolysis may affect the score). Other concerns arose when survival was not related primarily to hepatocyte failure, for example many of those with primary liver cell carcinoma were more likely to die or become ungraftable from their cancer. To address this problem, exception points were added so that the estimated prognosis by MELD more closely aligned to reality. Furthermore, other concerns were that MELD did not accurately reflect outcome in those with hepatopulmonary syndrome, portopulmonary hypertension, that those with some conditions such as primary sclerosing cholangitis had a prognosis not accurately reflected by the score. Finally, those with intractable symptoms, such as severe itch or encephalopathy, were not well served by the score. Children had their own score call the Pediatric model for end stage liver disease (PELD). This is applied to all children althouogh its validation is less robust than for MELD.

Concerns about equity persist. Geographical equity implies patients with similar severity of disease will have a similar chance of being offered a graft irrespective of where in the US they live, or which centre they are listed in. In practice, this goal is difficult to achieve in practice as the distribution of organ donors and recipients do not well match. In 2005, a regional share programme was introduced (Regional share 15) and was later expanded to status 1 patients in 2010, regional share 35 and national share 15 in 2013, and Acuity Circles in 2020. Share 35 rule is that patients with MELD-sodium score of 35 or above would be offered donated livers outside of the area of their Organ Procurement Organization (OPO) and within the same region. The Acuity Circles are designed further to reduce waiting list mortality, by trying to ensure the organ distribution is equal for listed candidates irrespective of where they live or wish to seek are listed. The older model based on the donor-service areas resulted in some patients being on more than one wait list travelling to other regions to get access to transplant. This model was challenged in the courts.

In the UK, the approach is slightly different. The jurisdiction is small, both in terms of population and geography; for a population of around 66 million, there are only seven liver transplant centres whereas New York (20 million) has 9 liver transplant centres and California (population 39 million) has 21 active liver transplant centres. Furthermore, in the UK with a nationalised health care service, transplant units are not in financial competition with each other. In the UK, the current policy is that the success of any national donor offering scheme would be judged on the basis of survival from the point of registration on a national list for a liver transplant, rather than the point of transplant (although data are given for both).

Allocation by need, or on the basis of utility, or by transplant benefit (net life years gained) were compared in a simulation against current unit-based allocation. A transplant benefit model was shown to reduce deaths on the waiting list and maximise population life years and since March 2018, liver donors after brain death in United Kingdom have been offered to a national list prioritised by net life years gained—transplant benefit (7, 72). In place of MELD, a UK based score was developed and is used the United Kingdom model for end stage liver disease (UKELD). This model, derived from the survival of patients on the UK transplant list was found to predict outcomes in these patients more accurately that MELD or its derivatives (Meld-Na). Similar to MELD-Na, this score includes serum sodium, INR, bilirubin and creatinine but the mathematical formula is different (73). At present, a UKELD score of 49 or above is the accepted threshold for being placed on the waitlist as this suggests an expected mortality of 9% at 1 year on the list which surpassed the 1-year post transplant mortality when this score was developed in 2011. In 2022, the 1-year post transplant mortality was 5% and therefore one could argue that this threshold for listing could be reduced (74). If the aim is to follow the “utility” principle, we would not advocate using the same model with limited factors (i.e., MELD, UKELD) to allocate organs as the effect they have on post-transplant outcome is different (73). In the UK, the Transplant Benefit Score is used to allocate grafts from brain death donors on a national basis. This 28 variable model (21 recipient and 7 donor) assigns a score for each donor-recipient match, and the recipients with the highest scores are sequentially offered the grafts. Minimal criteria for acceptance onto a transplant list have been developed for the situation of chronic liver disease with hepatocellular carcinoma, other than unresectability. The biggest issue in this situation is identifying those likely to experience cancer recurrence. In a number of variant syndromes where current scores do not adequately reflect the risk of death without a transplant or symptom burden (75).

Thus, while the MELD allocation system used by the US and the majority of other nations that use a predominantly deceased donor livers (76) is designed essentially to reduce mortality on the waiting list, the UK system is designed to ensure greatest survival benefit. Both goals are valid and ethical but will result in different selection and allocation processes. It is worth noting that neither approach incorporates quality of life of those on the waiting list or fully defines futility.

It should be stressed too that these are offering systems rather than “transplant” systems as centre decline rates vary greatly and this impacts on patient survival: Goldberg and colleagues analysed OPTN data of patients transplanted between 2007 and 2013, and included all adult liver-alone waitlist candidates offered an organ that was ultimately transplanted and found that among all patients ranked first on waitlists, the adjusted centre-specific organ acceptance rates ranged from 16% to 58% and the authors concluded that centre-level decisions to decline organs substantially increased patient's odds of dying on the waitlist without a transplant (77). No doubt a substantial part of this variation reflects the challenges of assessing the viability of the organ prior to implantation. The use of machine perfusion may provide the surgeon with more objective criteria on which to decide whether to use a donated organ (67).

Equity of access remains a major issue: a recent review from the US concluded racial and ethnic minorities, women, and patients in lower socioeconomic status groups were less likely to be referred, evaluated, and added to the waiting list for organ transplant (78). The situation in other jurisdictions is little better. Recognizing this inequity, the US Congress directed the National Institutes of Health to fund the National Academies of Sciences, Engineering, and Medicine in conducting a study on deceased donor organ procurement, allocation, and distribution, recommending ways to improve equity and accountability. Their report, Realizing the Promise of Equity in the Organ Transplantation System (79), agreed 14 recommendations for action that can be grouped into 3 areas: achieving equity, improving system performance, and increasing the utilization of available organs. Unfortunately, many of the recommendations were not new and aready subject to ongoing action and research. Goals such as developing objective measures to assess the viability of organs are hard to achieve in practice (80).

## 7. Future developments in liver transplantation

As is evident from the discussions above, the practice of liver transplantation has evolved at great pace over the last six decades and continues to change. Predicting the future is always hazardous and many predictions prove wrong. Nonetheless, we predict that in the immediate future, the place of machine perfusion in assessing the donated graft, maintaining and improving graft function will be established; we believe that methodologies will be developed to optimise perfusion techniques and provide robust biomarkers to help the surgeon decide which organs are acceptable (whether without or after interventions). In the longer term, we anticipate that immunosuppressive regimens will improve to allow for effective immunosuppression with further minimisation of adverse effects, although we note with concern the low probability of new agents reaching the clinic in the near future. The development of operational tolerance for all also seems a distant goal.

The donor organ shortage remains a challenge. Innovations to increase donation have generally limited success. Xenografts may be a solution in the longer term although there remain many immunological and physiological hurdles to be overcome. Other technologies may allow replacement of solid organ transplants such as use of autologous-derived cells on scaffolds or other technologies.

On the clinical side, we have seen how indications are evolving and it is clear that metabolic and alcohol associated liver disease will become major indications, with all the associated medical and other challenges associated with these conditions. As medical treatments for specific diseases and for the effect of fibrosis and cirrhosis become more effective, we hope that that the need for liver transplantation will reduce and patient's quality and quantity of life will be maintained or cured with less invasive means. Outcomes of liver transplantation remain sub-optimal and more attention needs to be paid to reduce the premature mortality of recipients and improve their quality of life.

One final concern we have is with the ability to maintain services. The provision of transplant services is very demanding on all members of the multi-disciplinary team but above all, there needs to be attention paid to ensuring that transplant surgery remains sufficiently enjoyable to attract and retain surgeons, without whom there would be no transplant.

## 8. Conclusion

In summary, outcomes with liver transplantation have greatly improved over the last 60 years and this has made it a viable treatment option for a broader range of conditions. The improved chance of longer-term survival following transplant has altered the risk-benefit balance in many new conditions in favour of the latter. These improvements cannot be attributed to a single factor and did not occur overnight. Rather, they reflect small incremental improvements in our understanding of the anaesthesia, surgical technique, perioperative care and the subsequent medical management of the transplant recipient. An improved understanding of organ donor and graft preservation factors has undoubtedly also had an impact in achieving optimal outcomes and expanding the donor pool. The patients that eventually become the recipients of a liver transplant only, represent a portion of individuals that would benefit from this treatment. This is largely due to the demand exceeding the supply of organs, but in some cases reflects inequity in access as a result to social factors.
